# Oral Lichen Planus: Clinical Presentation, Demographic Characteristics, and Risk Factors in a Retrospective Study of 186 Polish Patients

**DOI:** 10.3390/jcm13237363

**Published:** 2024-12-03

**Authors:** Zuzanna Ślebioda, Julia Drożdżyńska, Aleksandra Karpińska, Aleksandra Krzyżaniak, Marianna Kasperczak, Natalia Tomoń, Paulina Wiśniewska, Marzena Liliana Wyganowska

**Affiliations:** 1 Department of Dental Surgery, Periodontology and Oral Mucosa Diseases, Poznan University of Medical Sciences, 70 Bukowska St., 60-812 Poznań, Poland; 2 Medical Faculty, Poznan University of Medical Sciences, 60-812 Poznań, Poland

**Keywords:** oral lichen planus, oral mucosa, oral pathology

## Abstract

**Background/Objectives:** Lichen planus (LP) is a chronic, recurrent mucocutaneous inflammatory disease that develops due to a disturbed immunological response triggered by endogenous and exogenous factors. To evaluate clinical presentation, demographic characteristics, and risk factors in a cohort of Polish patients with oral lichen planus (OLP). **Methods:** Medical records of 186 patients with OLP referred to the student outpatient clinic in Poznań University of Medical Sciences from 2013 to 2023 were analyzed in order to establish clinical presentation, patient’s demographic characteristics, and risk factors for OLP. We considered data regarding age, sex, medical history, habits, subjective complaints, clinical type, localization, histology, and treatment. **Results:** Patients with OLP constituted 20.1% of 887 admitted patients. Of the 186 patients with OLP, 130 (69.9%) were females and 56 (30.1%) were males. The average age at the diagnosis was 60.7 years (range 15–95 years). Only 24.2% of patients reported smoking. The most common intraoral presentation of OLP was reticular form (61.8%), followed by mixed erosive-bullous type (15%), and atrophic (6.4%). Pathologic lesions were most commonly located on buccal mucosa (89.2%) and tongue (58.6%). Subjective complaints included pain (48.4%), burning sensation (46.2%), xerostomia (25.3%), bleeding (10.2%), taste disturbance (4.8%), and excessive saliva production (3.8%), while 22.6% of OLP patients were asymptomatic. **Conclusions:** The most common type of OLP in Polish patients was reticular, developing mostly on the buccal mucosa and tongue. It was found most often in non-smoking middle-aged women. Subjective complaints were reported by over 77% of patients.

## 1. Introduction

Lichen planus (LP) is a chronic inflammatory disease affecting skin and mucosal membranes. It develops due to a disturbed immunological response triggered by several endo- and exogenous factors and is characterized by recurrent episodes [1,2]. Although the etiology of LP has not been established, it is generally accepted to be a T-cell-mediated condition [1,2,3,4,5].

LP may affect the skin and mucous membranes. Skin lesions appear as itchy, flat, pink papules. Most common intraoral lesions present as white, symmetrically disturbed papules, forming reticular or plaque-like networks with a bilateral location, often accompanied by erosive, erythematous, or bullous eruptions. All types of OLP can be classified into two main clinical variants: erosive-atrophic (EA-OLP), and non-erosive-atrophic forms (non-EA-OLP). The first group comprises reticular, plaque, and papular subtypes; while the second group includes erosive, atrophic, bullous, and mixed erosive-atrophic variants [1,3,6,7,8]. The coincidence of skin and mucosal lesions is common, with frequency from 4% to 44%. The oral lichen planus occurs more often than the cutaneous and shows higher resistance to treatment [1,2,5].

The prevalence of OLP in the general adult population ranges between 0.5% and 2% [1,2]. The average age of onset is estimated between 30 and 60 years, with a female predilection [1,7], and the development of the disease in children is infrequent [1,2].

According to the World Health Organization (WHO), OLP belongs to oral potentially malignant disorders (OPMD) with a transformation risk from 0.4% to 3.3% throughout 0.5 to >20 years, more commonly deriving from atrophic-erosive types [8]. The mechanisms of carcinogenesis in OLP have still not been fully elucidated. OLP development and progression from non-EA into EA types may be stimulated by virobiota, namely hepatitis C virus (HCV), herpesvirus (HSV), and human papillomavirus (HPV) [1,2,7]. 

The treatment of OLP is mainly symptomatic, and an effective causative strategy has yet to be developed. The most commonly used drugs include corticosteroids. Alternatively, calcineurin inhibitors, azathioprine, mycophenolate mofetil, retinoids, dapsone, and hydroxychloroquine can be considered [7].

Several epidemiological studies of OLP have described the population of the United States, Asia, and some European countries [9,10,11,12,13,14,15,16,17]. The reports on the demographic and clinical presentations of OLP in Polish patients are limited and include relatively small numbers of patients [18,19,20,21]. Therefore, further topic analysis seems required.

This study aims to perform a retrospective analysis of clinical presentation, basic demographic features, and potential risk factors of a cohort of 186 Polish patients with OLP. This study expands the knowledge of the epidemiological characteristics of OLP patients from Northeastern Europe.

## 2. Materials and Methods

We performed a retrospective chart review of patients treated at the Department of Dental Surgery, Periodontology and Oral Mucosa Diseases, Poznań University of Medical Sciences, from 1 January 2013 to 31 December 2023. The study group comprised 186 patients diagnosed with OLP referred to the student outpatient clinic, constituting 20.1% of the 887 analyzed records. Data regarding age, sex, chief complaint, clinical type of OLP, localization, histology, and treatment were registered. Furthermore, information on medical history, drugs, smoking, and alcohol consumption was collected. The diagnosis of OLP was based on clinical criteria with a biopsy performed in atypical or refractory cases, and histopathological criteria according to WHO were used [22,23]. OLP was classified into the following types: reticular, characterized by the presence of nonremovable, lacelike keratotic lesions on the oral mucosa; bullous-erosive (mixed), with well-defined erosions or bullae; atrophic, with the regions of thinned/atrophic epithelium within previously defined keratotic lesions; desquamative (exfoliative) gingivitis, with the erythematous gingiva, desquamation and erosion of the gingival epithelium, and blister formation; and plaque-like, with a dense thickening of the mucosal tissue.

Data were organized in MS Excel^®^ spreadsheets and presented descriptively. Mann–Whitney, Fisher’s exact, chi-square tests, and the difference test between two proportions were applied where appropriate, with *p* values lower than 0.05 considered significant. We used Dell Statistica (data analysis software system), version 13 (Dell Inc., 2016; Palo Alto, CA, USA). 

## 3. Results

Essential demographic characteristics of the study group are presented in Table 1 and Table 2. 

We reviewed the medical records of 186 patients diagnosed with oral lichen planus, of whom almost 70% were females (significant difference; *p* < 0.0001). Significantly more of the study participants reported urban residence (66.6%). The mean age of the participants was 60.8, and it was higher in females than in males, though the difference was insignificant. The majority of patients represented the age group 56–75 (53.8% of subjects), while the fewest number of patients were aged 15–35 (10; 5.4%). A significant difference in OLP frequency between females and males was revealed only in the third (56–75 years old) group of participants.

The summary of the most commonly observed clinical types of OLP in relation to sex, habit of smoking, and age of the study participants is presented in Table 3. 

For this analysis, we distinguished the following types of OLP: reticular, mixed bullous-erosive, atrophic, exfoliative (desquamative gingivitis), and plaque-like. Based on the records, the reticular type was detected most frequently (122 cases; 65.6%). It was found slightly more often in females than males, and the difference was not statistically significant. Moreover, we did not reveal any sex predilection to any particular type of OLP confirmed in statistical analysis. There was also no predilection to any OLP subtype regarding smoking or age group. It was found that 45 (24.19%) of all patients (186) diagnosed with OLP admitted to smoking, one of them (0.54%) was addicted to alcohol, and two patients (1.08%) reported being addicted to both alcohol and smoking. In general, OLP was found significantly more often in non-smokers compared to smokers (*p* < 0.0001), although no significant differences regarding the OLP subtypes and smoking were revealed.

**Figure 1:**
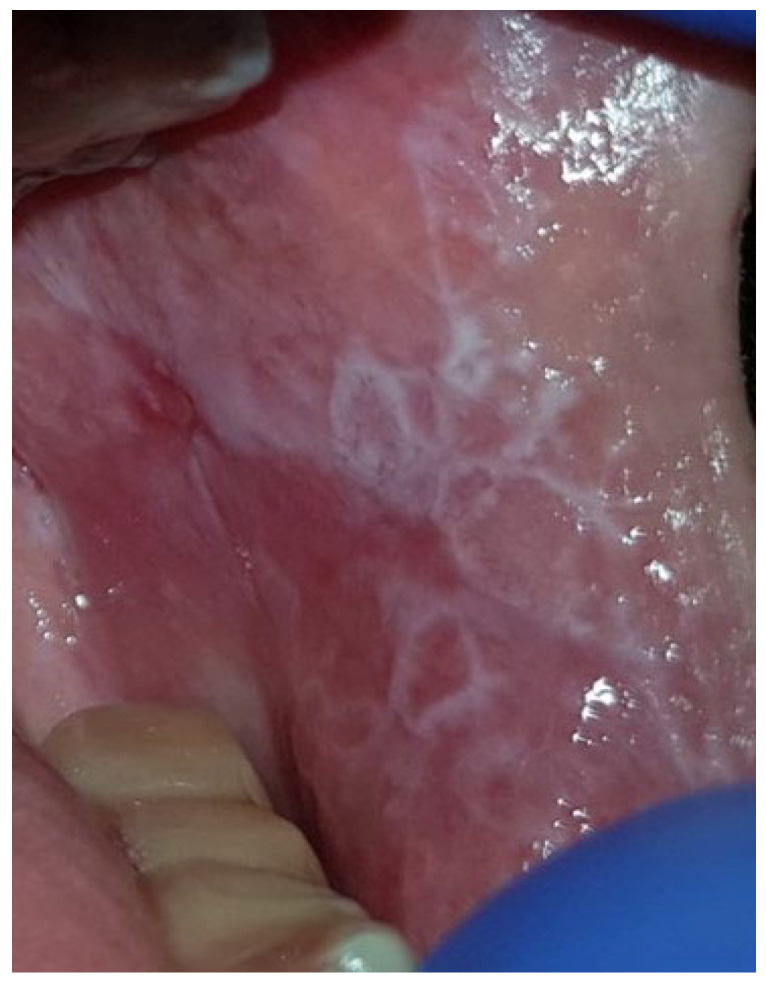
Reticular LP with characteristic Wickham striae on the buccal mucosa.

**Figure 2:**
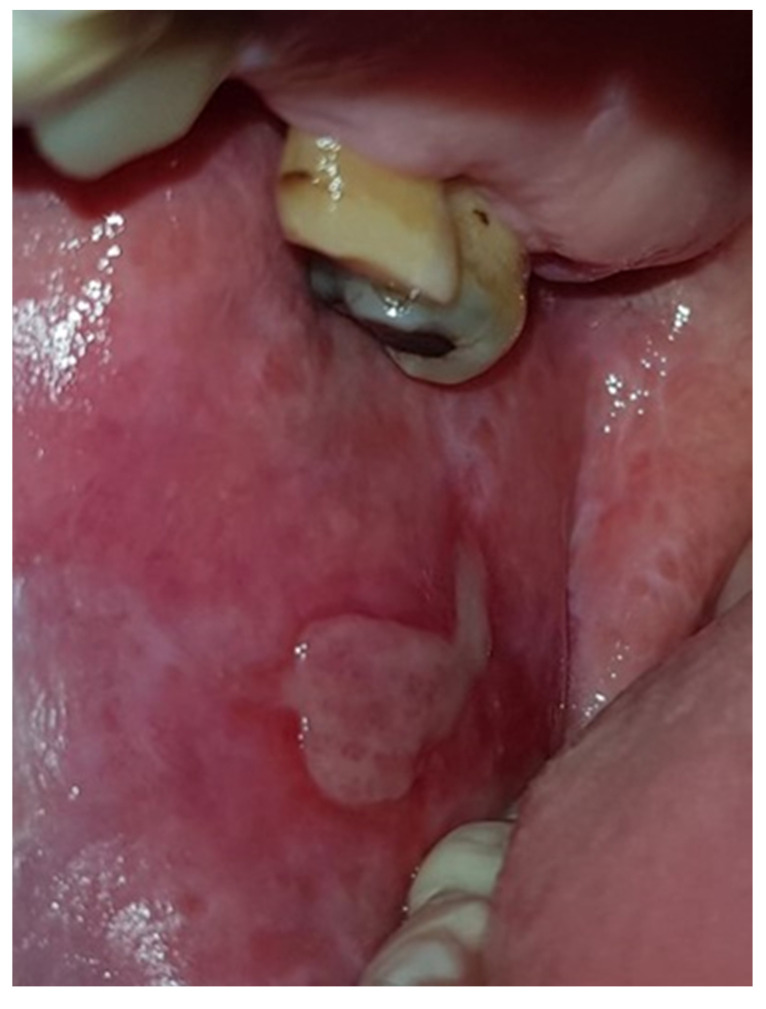
Erosive-bullous form of OLP on buccal mucosa.

**Figure 3:**
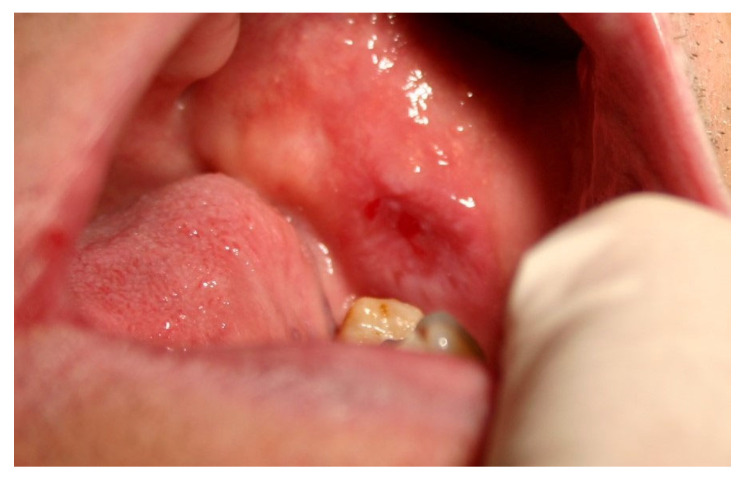
Atrophic form of OLP on buccal mucosa.

Figure 1, Figure 2 and Figure 3 depict reticular, mixed (erosive-bullous), and atrophic forms of OLP.

Subjective symptoms were reported by 77.4% of all study participants, and some of them described more than one complaint at one time. The most commonly reported complaints included pain, burning, and xerostomia. Other occasionally reported subjective symptoms were bleeding from the oral mucosa, taste disturbances, and excessive saliva production. Table 4 illustrates the number and type of subjective symptoms in the study population.

Histopathologic examination was performed on 40 patients from the study group. The results are presented in Table 5 and Table 6. In eight subjects, features consistent with OLP without atypia or dysplasia were revealed. Other common diagnoses included nonspecific inflammation and para- or hyperkeratosis without atypia (seven patients, respectively). Inflammatory granulation tissue was revealed in three cases. The mild-stage dysplasia was also found in three subjects. We detected single cases of keratinizing squamous cell carcinoma (stages G1 and G2). One case was related to reticular, and one was derived from an atrophic OLP form.

**Figure 4:**
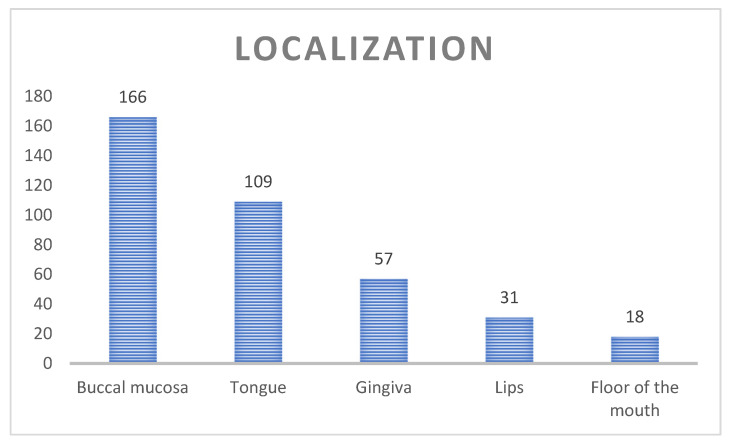
Localization of the lesions in the oral cavity.

Figure 4 depicts the localization of the oral lesions typical for OLP in the study group. In several cases, more than one location was observed for one patient. Most commonly, the buccal mucosa was involved (166 patients). The tongue was affected in 109 cases, while desquamative gingivitis was revealed in 31 subjects. Less frequently, the labial mucosa and floor of the mouth were involved (31 and 18 patients, respectively). In 38 patients (20.4%), skin lesions typical for LP were also detected.

Twenty-one patients from the study group (11.2%) were referred to our outpatient clinic by other specialists. That included dermatologists (five cases), dentists (four cases), laryngologists, and maxillofacial surgeons (one case, respectively). 

Table 7 and Table 8 depict systemic diseases revealed in the study group. Over 80% of the study population with OLP reported at least one systemic condition; of those the most frequently reported were cardiovascular disorders (89; 47.8%), disorders of the thyroid gland (38 cases; 20.4%), and gastrointestinal diseases (30; 16.1%). Type 2 diabetes was revealed in 18 cases (9.7%). Our study group had no statistically significant predilection towards any OLP subtype regarding systemic conditions. 

Local treatment of oral LP lesions was utilized in 155 patients (83.3%). The most common therapeutic strategy included herbal coating mouth-rinse, composed of flax seed, chamomile, mallow, and marshmallow, mixed in equal proportions (94 patients). The seven other patients used ready-to-use mouth rinse composed mainly of flax seed, while linseed oil was applied directly to the oral mucosa and administered to 10 patients. Topical steroids were administered in 43 cases and included dexamethasone (37), clobetasone (3), hydrocortisone (2), and fluocinolone (1). None of the patients was qualified for systemic treatment with steroids. Calcineurin inhibitors were applied in nine patients. Topical vitamin A was used in 12 cases, and low-level laser therapy was utilized in 16 patients. 

## 4. Discussion

Based on our observation, OLP appeared most frequently in middle-aged and older women, which aligns with several other authors’ reports [9,10,14,15,17,20,24,25,26,27]. We showed a significant female predilection for OLP in general, although the patients’ sex did not promote any particular OLP subtype. Contrary results were shown by Bandyopadhyay et al., who found insignificant male predilection in OLP (54.55% males vs. 45.45% females) [28] and in the Munde et al. study, where the M:F ratio was 1.61:1 [29]. As mentioned in Gorouhi’s review, there is no evident sexual predilection for OLP. However, a slight predominance of female patients is commonly reported in studies on adults, while males were affected more often in childhood cases of OLP [30]. That was described by Sharma et al., Karma et al., and Walton et al. [31,32,33]. For many autoimmune diseases, there is a solid female predilection, as sex hormones play an essential role in autoimmunity regulation. Estrogens are considered to be potent stimulators of autoimmunity, while androgens play a protective role. Moreover, sex-related differences in the prevalence and the course of autoimmune diseases are modified by genetic, epigenetic, and environmental factors [34]. That includes the microRNA expression, the role of sex chromosomes, sex-related gut microbiota variations, microchimerism, and the influence of exogenous estrogens [34]. Sex-related diversity of microRNA expression, crucial in the development and function of innate and adaptive immune cells, was observed in both gonadal and non-gonadal tissues.

Meanwhile, overexpression of several immune-related genes encoded by the X chromosome, such as CD40 ligand, chemokine receptor CXCR3, toll-like receptor (TLR)7, TLR8, IL-2 receptor gamma, tyrosine-protein kinase BTK, and IL-9 receptor, may affect the immune system action in a sex-dependent manner [34,35]. On the one hand, hormonal changes typical for pregnancy in several ways prevent pregnant individuals from becoming ill and enable the development of the fetus. On the other hand, many periodontal diseases exacerbate during pregnancy, making oral mucosa more vulnerable. Weakened tissue and repetitive micro-damage may promote reoccurring inflammation [36]. The relative male predilection of OLP in children suggests that other than autoimmune mechanisms, it plays a role in the pathogenesis of LP [30]. In our observations, the female:male ratio, which is 2.3:1, supports the thesis of the autoimmune background of the condition. 

As mentioned in our study, the peak incidence of OLP was observed in patients who reached middle- to late-life stages. The mean age was 60.8 years, and the youngest patient was 15, but that was a single case of a preadult person in our study group. Most of the patients represented the age group 56–75 (53.8%). That is again in agreement with studies of several authors [9,10,13,15,20]. No child with OLP was observed in the retrospective survey of Ingafou et al. from the UK [9]. Although childhood cases of OLP were described by the reports of Sharma et al., Karma et al., and Walton et al., the results of most epidemiological studies, as with our study results, reflect the rarity of OLP in childhood [31,32,33]. The prevalence of OLP in children is reported to be 0.03% [10]. Contrary to our results, in the studies of Pakfetrat et al., Bandyopadhyay et al., and Vijayan et al., the peak incidence of OLP was observed in the younger age groups (30–44 years, 28–37 years, and 25–34 years, respectively) [15,27,28]. In agreement with similar studies, OLP developed at similar ages in both sexes in our study population. According to several authors, there is a tendency for all types of OLP to appear at an earlier age in males than in females [9,14]. In our series, the mean female age was also higher than the mean male age; the difference was not statistically significant. 

In this study, a reticular type of OLP was detected most frequently. It was found slightly more often in females than males, but the difference was not statistically significant. Moreover, we did not reveal any sex predilection to any particular type of OLP confirmed in the statistical analysis. There was also no predilection to any OLP subtype regarding smoking or age group. That aligns with the study by Thum-Tyzo et al., where no predominance of a particular form of oral lichen planus, neither to the age of patients (*p* = 0.52), nor to the sex of patients (*p* = 0.73) was found [20]. The majority of the reports on OLP declare the reticular form as the most commonly observed, with a frequency of 58% [27], 61.5% [24], 83.5% [29], 88.7% [20], and 95.6% [13] according to different sources. Contrary to these observations, Bermejo-Fenoll found the erosive-atrophic form of OLP to be the leading type in their study group (65.3%) [14]. Similar to our results, in the Kesic et al. study, the reticular (*p* < 0.05) and atrophic (*p* < 0.01) types of OLP were predominant in women [37].

A total of 77.4% of the study participants reported subjective symptoms, including pain, burning, xerostomia, bleeding from the oral mucosa, taste disturbances, and excessive saliva production. The most common chief complaint in the Budimir study was oral soreness, reported by 43.3% of patients. The prevalence of subjective complaints was lower than in our research and reached 55.8% [10]. Thum-Tyzo et al. observed symptomatic changes in nearly all examined subjects with OLP (96.2%). That included dryness (62.26%), burning in the oral cavity (52.83%), and numbness of the region affected by lesions (49.06%) [20]. In general, the ratio of asymptomatic patients distinctly varied depending on the authors, and the levels reached were between 16% and 60% [9,10,14,15]. Such differences can be partially explained by a recurrent mode of the condition with periods of exacerbations, where the symptoms are more evident, and remissions, when the complaints become reduced. Another explanation is the unstandardized approach to evaluating the subjective complaints used in different research centers. Nevertheless, our observations, where over 70% of the examined subjects suffered some complaints, show that OLP may severely impair the patient’s quality of life in a prolonged manner. 

In cases of evident clinical features of OLP, the diagnosis is often formed on this basis. All atypical cases and cases that do not respond to a conservative approach to treatment require histopathological confirmation. Cheek chewing/frictional keratosis, lichenoid reactions, leukoplakia, lupus erythematosus, pemphigus, mucous membrane pemphigoid, erythematous candidiasis, and chronic ulcerative stomatitis are the most common conditions to be considered in the differential diagnosis [10]. Based on retrospective data from our department, a biopsy was performed in 21.5% of OLP subjects when the diagnosis was not straightforward. Our study group’s most common histopathologic findings included features consistent with OLP without atypia or dysplasia, nonspecific inflammation, and para- or hyperkeratosis without atypia. Mild-stage dysplasia was found in two subjects (one with bullous-erosive OLP and one with plaque-like OLP), while a focal atypia/dysplasia was revealed in one patient with plaque-like OLP. Single cases of keratinizing squamous cell carcinoma (stages G1 and G2) were also detected. Surprisingly, one case was related to the reticular OLP type. The other one was derived from an atrophic OLP form. According to several sources in the literature, malignant transformation is mostly related to mixed, bullous-erosive forms or red types of OLP, like atrophic or erosive [1,2,8,10]. As concluded by Tsushima et al., based on the data derived from recent meta-analyses, tongue localization, tobacco and alcohol consumption, and HCV infection significantly heighten the risk of the malignant transformation of OLP [17]. In their observations, for the age of 62 years and higher, gingiva, and red-type OLP tended to have a higher risk of squamous cell carcinoma (SCC), found in 0.7% of their patients. Three of these four patients represented the red type of OLP [17]. That aligns with the fact that the rate of malignant transformation of OLP is relatively low and estimated to be between 0.07% and 5.8% [1,2,9,10,14,37]. However, this risk should be considered, and careful monitoring of all OLP patients is advised. The mechanisms of carcinogenesis in OLP have not been fully explained. However, a potential role stimulation with virobiota, namely hepatitis C virus (HCV), Epstein–Barr virus, herpesvirus (HSV), cytomegalovirus (CMV), and human papillomavirus (HPV), was suggested [1,2,7]. 

In this study group, the most common location of the oral lesions was the buccal mucosa, followed by the tongue. Desquamative gingivitis was revealed in 31 (16.7%) subjects. Following several other reports, as in most studies, the buccal mucosa is the most common site of OLP and is reported to be affected in 73% to 95.5% of the patients [9,10,15,26]. Desquamative gingivitis, a descriptive term that denotes the presence of erythema, desquamation, erosion, and blistering of the gingival epithelium, is considered a nonspecific clinical response of the gingiva to various mucocutaneous conditions. About 75% of reported cases have a dermatological origin, caused mainly by lichen planus, mucous membrane pemphigoid, and pemphigus vulgaris [38]. In 38 patients (20.4%), skin lesions typical for LP were also detected. Similar results were revealed in the Thum-Tyzo et al. study, where 22.6% of OLP subjects had similar skin lesions [20]. The coincidence of skin and mucosal lesions in LP is common, although the rate of this phenomenon varies from 4% to 44%, depending on the authors. The oral form of lichen planus occurs more frequently and tends to be more resistant to treatment than the cutaneous type [1,2,5].

Over 80% of the study population with OLP reported at least one systemic condition; of those most frequently reported were cardiovascular disorders (89; 47.8%), disorders of the thyroid gland (38 cases; 20.4%), and gastrointestinal diseases (30; 16.1%). Type 2 diabetes was revealed in 18 cases (9.7%). A similar frequency of type 2 diabetes was found in the study by Carbone et al. (8.1%) [39]. In the Thum-Tyzo et al. study, the frequency of the reported systemic diseases in the OLP group reached 71.7%, although the distribution was different than in our observations; as in our study, cardiovascular diseases were the most frequent (54.72%), while diseases of the gastrointestinal, respiratory, and nervous systems constituted a small part of the revealed systemic conditions. In their study, type 2 diabetes occurred in 11.32% [20]. A much lower frequency of systemic diseases among OLP patients was revealed in the Bandyopadhyay study, where about 68.53% did not report systemic conditions. Of the remaining 31.47%, type 2 diabetes mellitus (DM) was the most common (13.29%), followed by the concomitant presence of both Type 2 DM and hypertension (8.39%) [28]. A triad composed of OLP, type 2 diabetes, and hypertension is called Grinspan syndrome, which remains an enigmatic condition. It is either a separate entity or a drug-induced lichenoid reaction emerging due to medications used to treat hypertension and diabetes [40]. The thyroid gland disorders, the second most commonly observed systemic condition accompanying OLP in our research, are often of an autoimmune origin. Concomitant development of at least three autoimmune diseases in the same patient has been defined as multiple autoimmune syndrome (MAS). Nearly 25 percent of patients with one autoimmune disease tend to develop additional autoimmune disorders [41]. Our study group had no statistically significant predilection towards any OLP subtype regarding systemic conditions. 

From all the referrals, dermatologists and dentists were the largest group suggesting patients visit an oral pathology department. This dependence can result from the nature of the disease, as both oral and skin lesions may occur. 

We found that 45 (24.19%) of all patients (186) diagnosed with OLP admitted to smoking, one (0.54%) was addicted to alcohol, and two patients (1.08%) reported being addicted to both alcohol and smoking. In our study, OLP was found significantly more often in non-smokers compared to smokers (*p* < 0.0001), although no significant differences regarding the OLP subtypes and smoking were revealed. Smoking and alcohol drinking were also not common among OLP patients in the Budimir et al. study, where 78.4% of patients were non-smokers, and 70.9% of the patients did not drink alcohol excessively [10]. Those habits, therefore, do not seem to play an essential role in the development of OLP, as OLP patients have no increased prevalence of smoking or alcohol abuse compared to the general population [10,26]. Those results are somewhat surprising, considering the well-documented harmful effects of tobacco and exposition to alcohol. It promotes oxidative stress and stimulates an aggravated immune response. Tobacco smoke and its cancerous elements induce microbiome changes and disrupt tissue hemostasis [42]. According to some authors, smoking and alcohol consumption are also not associated with an increased risk of malignant transformation in OLP [10,14,17].

In our study, some treatment was administered to over 80% of OLP patients. As the etiology of OLP is unclearly defined, no causative treatment is available [1,2,10]. The common complaint reported by OLP patients is pain. This indicates a genuine concern and proves that this specific disease entity should be analyzed from the pain relief perspective. Treatment strategies, therefore, should focus on relieving the symptoms and reducing the functional impact of the disease. As the background of the condition is related to exacerbated inflammatory response, immunosuppressive drugs are commonly used in exacerbated types of OLP. In our report, topical corticosteroids were administered to 27.5% of the study group, while calcineurin inhibitors were used in 4.8% of patients. In Radochová et al., topical steroids alone or in combination with systemic steroids were prescribed more often than in our unit. Approximately 70% of their study population received that kind of treatment; as in our setting, dexamethasone gel was applied several times daily to the most symptomatic areas. A Czech study also used a combination of dexamethasone gel and a depot form of corticosteroid intralesionally [12,13]. We did not introduce the intralesional approach in our research. Common supportive treatment strategies in our study population included topical administration of flax seed (linseed) containing mouth rinses. Linseed is a plant that forms a colloidal solution, which, when administered orally, does not penetrate through the mucous membrane, but covers it, creating a protective layer. It preserves the mucous membrane from damage induced by various agents, bacterial toxins, medicines, and mechanical injuries, and moistens the oral cavity [43].

## 5. Limitations of the Study

This study is a retrospective observational survey and, as such, it has many limitations. That includes an ununiform diagnostic approach, limited control of population sampling, and limited control over the nature and quality of the predictor variables. That obviously may interfere with the precision of the obtained results. Nevertheless, we believe that this analysis is valuable, as it shows new data on the Polish cohort of OLP patients. 

## 6. Conclusions

This study sheds additional light on the epidemiological characteristics of OLP patients from Northeastern Europe. Most of the characteristics are consistent with previous studies on OLP from various geographic regions. The results of this study reveal that Polish patients with OLP are typically middle-aged women of urban origin, with oral lesions located mainly on the buccal mucosa, tongue, and gingivae. The most commonly observed type is reticular. Extraoral involvement accompanies the oral signs relatively rarely. OLP develops more often in non-smokers than in smokers and is commonly accompanied by cardiovascular diseases. Age, sex, habits, and comorbidities do not promote any particular OLP subtype. Although the risk of malignant transformation is low, careful monitoring by qualified clinicians is recommended for OLP patients due to a chronic course and unpredictable mode of recurrences.

## Figures and Tables

**Table 1:** Demographic characteristics of the study group, considering sex distribution, residence, and smoking.

	Number of Patients N (%)	*p*
Total	186 (100%)	
Sex distribution		
Females	130 (69.9%)	<0.0001
Males	56 (30.1%)	
Residence		
Rural	62 (33.3)	<0.0001
Urban	124 (66.6)	
Habits		
Smokers	47 (25.2)	<0.0001
Non-smokers	136 (74.8)	

**Table 2:** Age groups of the study participants in relation to sex.

	Total	Females	Males	*p*
Age (mean ± SD, range [years])	60.8 ± 14.7 15–95	61.9 ± 20.5 15–88	57.9 ± 15.6 29–95	0.1933
Age groups	N (%)			
15–35	10 (5.4)	6 (3.2)	4 (2.1)	0.5090
36–55	51 (27.4)	31 (16.7)	20 (10.7)	0.0924
56–75	100 (53.8)	76 (40.9)	24 (12.9)	<0.0001
76–95	25 (13.4)	18 (9.7)	7 (3.8)	0.0233

**Table 3:** Clinical presentation of OLP in the study group in relation to sex, habit of smoking, and age of the study participants.

The Clinical Form of OLP	Total (N = 186)	Females (N = 130)	Males (N = 56)	*p*	Smokers (N = 47)	Non-Smokers (N = 139)	*p*	Age Groups			*p*
								<46 (N = 30)	46–75 (N = 131)	>75 (N = 25)	
Reticular	122 (65.6%)	87 (66.9%)	35 (62.5%)	0.5623	33 (70.2%)	89 (64.0%)	0.4393	20 (66.7%)	86 (65.5%)	16 (64.0%)	0.9784
Bullous/Erosive (Mixed)	36 (19.4%)	23 (17.7%)	13 (23.2%)	0.3838	8 (17.0%)	28 (20.1%)	0.6417	5 (16.7%)	25 (19.1%)	6 (24.0%)	0.7825
Atrophic	13 (7.0%)	11 (8.5%)	2 (3.6%)	0.2303	3 (6.4%)	10 (7.2%)	0.8526	1 (3.3%)	10 (7.7%)	2 (8.0%)	0.6908
Exfoliative (desquamative gingivitis)	11 (5.8%)	7 (5.4%)	4 (7.1%)	0.6520	2 (4.3%)	9 (6.5%)	0.5813	3 (10.0%)	8 (6.2%)	0 (0.0%)	0.6224
Plaque-like	4 (2.2%)	2 (1.5%)	2 (3.6%)	0.3631	1 (2.1%)	3 (2.2%)	0.9676	1 (3.3%)	2 (1.5%)	1 (4.0%)	0.6544

**Table 4:** Subjective complaints in study participants with OLP.

Subjective Complaint	n, % of TSP *
Number of patients reporting complaints	144 (77.4%)
Number of patients not reporting any complaints	42 (22.6%)
Total number of complaints	334
Pain	90 (48.4%)
Burning sensation	86 (46.2%)
Xerostomia	47 (25.3%)
Bleeding	19 (10.2%)
Taste disturbances	9 (4.8%)
Excessive saliva production	7 (3.8%)
Other	76 (40.9%)

* TSP—total study population (186).

**Table 5:** Results of histopathologic examination of the OLP patients.

Histopathological Evaluation (*n* = 40)	n
Consistent with OLP without atypia or dysplasia	8
Parakeratosis/hyperkeratosis without atypia	7
Nonspecific inflammation	7
Inflammatory granulation tissue	3
Mild dysplasia	3
Erosion/Ulcer	2
Fibroma	2
Papilloma	2
Nonspecific inflammation with acanthosis	2
Keratinizing squamous cell carcinoma (stage G1)	1
Keratinizing squamous cell carcinoma (stage G2)	1
Other	2

**Table 6:** Histopathological findings in relation to patients’ age, sex, comorbidities, and habits.

Patient	Age	Sex	Clinical OLP Type	Comorbidities	Habits	Histopathologic Findings
SB	63	M	Reticular	Hypertension, Urinary tract disorders	Smoking	Hyperkeratosis and inflammation
TU	42	M	Reticular	NR	Smoking	Parakeratosis without atypia
KH	67	F	Reticular	Hypertension, Gastritis	Smoking	Nonspecific inflammation with acanthosis
IB	53	F	Reticular	Hypertension, Obesity	NR	Keratinizing squamous cell carcinoma (stage G1)
IM	70	F	Reticular	Hypertension, Parkinson’s disease, Brain stroke	Smoking	Para- and hyperkeratosis without atypia, inflammation
ES	49	F	Reticular	NR	Smoking	Squamous papilloma
DZ	70	F	Reticular	Hypertension	NR	Squamous papilloma
KW	67	F	Reticular	Epilepsy, Hypothyroidism, Depression	NR	Hyperkeratosis
IW	52	F	Reticular	Hypertension, Depression	Smoking	Para- and hyperkeratosis, inflammation without atypia
EW	73	K	Reticular with desquamative gingivitis	Parkinson’s disease, Asthma, Hypertension, Diabetes type II, Urinary tract and lung cancer	NR	Fibroma
RS	71	M	Bullous-erosive	Hypothyroidism, Psoriasis, Ischaemic heart disease	NR	Spongiosis
SL	84	M	Bullous-erosive	Hypercholesterolaemia	NR	Para- and hyperkeratosis, inflammation with mild focal dysplasia
KK	85	F	Bullous-erosive	Hypertension, Rheumatoid arthritis	NR	Chronic inflammation with erosion
EF	57	K	Bullous-erosive	Hypertension, Rheumatoid arthritis	NR	Spongiosis, erosions, inflammatory granulation tissue
MS	41	F	Bullous-erosive	NR	NR	Inflammatory granulation tissue
LK	68	M	Bullous-erosive	Hypertension, Insulin resistance, Vitiligo, Allergy to pollen	NR	Consistent with OLP without atypia or dysplasia
MA	67	M	Plaque-like	Hypertension, Chronic Obstructive Pulmonary Disease	NR	Consistent with OLP without atypia or dysplasia
KR	37	F	Plaque-like	Insulin resistance, Depression	Smoking	Akantosis, para- and orthokeratosis without dysplasia, inflammation, ulceration
MSZ	47	F	Plaque-like	Asthma	Smoking	Parakeratosis, inflammation
BW	75	F	Plaque-like	Hypertension, Nephritis, Hepatic steatosis	NR	Squamous papilloma; Focal atypia/dysplasia
KN	58	F	Plaque-like	Hypertension, Gastric cancer, Venous thrombosis, Psoriasis	Smoking	Inflammatory granulation tissue with mild dysplasia
DK	59	K	Atrophic	Hypertension, Epilepsy, Depression	NR	Keratinizing squamous cell carcinoma (stage G2)
RG	60	K	Atrophic	Gastro-oesophageal reflux disease	NR	Fibroma
SN	85	F	Atrophic	Hypertension, Colon cancer, Sleep disorders	NR	Chronic inflammation with no signs of immunobullous disease
EK	30	F	Atrophic	NR	NR	Consistent with OLP without atypia or dysplasia

NR—not reported.

**Table 7:** Systemic diseases in the study group.

Systemic Diseases	n
Disease reported	152 (81.7%)
Disease not reported	34 (18.3%)
Cardiovascular diseases	89 (47.8%)
Thyroid disorders	38 (20.4%)
Gastrointestinal diseases	30 (16.1%)
Type 2 diabetes	18 (9.7%)
Neurological diseases	12 (6.4%)
Other	90 (48.4%)

**Table 8:** Systemic diseases in the study group in relation to the OLP types.

	Thyroid Gland Disorders	Type II Diabetes	Cardiovascular Disorders	*p*
OLP	38	18	89	
Reticular	27	12	55	0.7647
Bullous/Erosive (Mixed)	5	3	21	0.4192
Atrophic	1	1	10	0.2623
Exfoliative (desquamative gingivitis)	3	1	2	0.3332
Plaque-like	2	1	1	0.3217

## Data Availability

All data underlying the results are available from the corresponding author upon request.
